# Changes in corticosteroid and non-steroidal immunosuppressive therapy with long-term zilucoplan treatment in generalized myasthenia gravis

**DOI:** 10.1007/s00415-025-13113-0

**Published:** 2025-06-12

**Authors:** Channa Hewamadduma, Miriam Freimer, Angela Genge, M. Isabel Leite, Kimiaki Utsugisawa, Tuan Vu, Babak Boroojerdi, Fiona Grimson, Natasa Savic, Mark Vanderkelen, James F. Howard, on behalf of the RAISE-XT study team

**Affiliations:** 1 Academic Neuromuscular Unit, Sheffield Teaching Hospitals NHS Foundation Trust, Sheffield, UK; 2 Sheffield Institute for Translational Neurosciences (SITraN), University of Sheffield, Sheffield, UK; 3 Department of Neurology, The Ohio State University Wexner Medical Center, Columbus, OH USA; 4 Clinical Research Unit, The Montreal Neurological Institute, Montreal, QC Canada; 5 Nuffield Department of Clinical Neurosciences, University of Oxford, Oxford, UK; 6 Department of Neurology, Hanamaki General Hospital, Hanamaki, Japan; 7 Department of Neurology, University of South Florida Morsani College of Medicine, Tampa, FL USA; 8 UCB, Monheim, Germany; 9 UCB, Slough, UK; 10 UCB, Bulle, Switzerland; 11 UCB, Brussels, Belgium; 12 Department of Neurology, The University of North Carolina at Chapel Hill, Chapel Hill, NC USA

**Keywords:** Myasthenia gravis, Corticosteroid sparing, Immunosuppressive therapy, Zilucoplan

## Abstract

**Background:**

The efficacy and safety of complement component 5 inhibitor zilucoplan in patients with anti-acetylcholine receptor antibody-positive generalized myasthenia gravis (gMG) were assessed in two double-blind studies (NCT03315130/NCT04115293 [RAISE]). During these studies and the first 12 weeks of the open-label extension study, RAISE-XT, corticosteroid and non-steroidal immunosuppressive therapy (NSIST) doses were kept stable; thereafter doses could be changed at the investigator’s discretion. We evaluated corticosteroid and NSIST dose changes in patients with gMG during zilucoplan treatment in RAISE-XT.

**Methods:**

In RAISE-XT, patients who completed a qualifying double-blind study self-administered once-daily subcutaneous zilucoplan 0.3mg/kg. We assessed (post hoc) patients who changed their corticosteroid or NSIST dose relative to double-blind baseline at Week 120 (data cutoff: November 11, 2023).

**Results:**

Overall, 200 patients enrolled. At Week 120, 61.1% (n = 33/54) of patients who were on corticosteroids at double-blind baseline had reduced or discontinued corticosteroids (mean 15.5mg dose reduction); mean change from baseline (CFB) in Myasthenia Gravis Activities of Daily Living (MG-ADL) score:−6.55 (standard deviation [SD] 3.65). Of patients on NSIST at double-blind baseline, 29.8% (n = 14/47) reduced or discontinued ≥ 1 NSIST; mean CFB in MG-ADL score:−7.57 (SD 4.69). Among all patients at Week 120, 9.3% (n = 8/86) had increased or started corticosteroids; 2.4% of patients (n = 2/85) had increased NSIST, including one who started a new NSIST. Zilucoplan was well tolerated.

**Conclusions:**

Treatment with zilucoplan allowed for reduction or discontinuation of corticosteroids in the majority of patients and NSIST in about a third of patients, while maintaining efficacy.

**Trial registration:**

NCT04225871; October 2, 2019.

**Supplementary Information:**

The online version contains supplementary material available at 10.1007/s00415-025-13113-0.

## Introduction

Myasthenia gravis (MG) is an autoimmune disease characterized by exertional muscle fatigue and weakness of skeletal muscles due to impaired neuromuscular transmission and architectural destruction of the neuromuscular junction [1, 2]. The international consensus guidance for the management of MG recommends corticosteroids (CS) as a first-line immunotherapy for patients who have not met treatment goals with acetylcholinesterase inhibitors [3]; oral prednisone and prednisolone are widely used for this purpose [1, 4]. Non-steroidal immunosuppressive therapy (NSIST), such as azathioprine and cyclosporine, is often used in conjunction with CS [1, 4]. Early treatment with CS may lead to early and long-term remission in patients with MG, with 72–82% of patients who receive CS achieving improvements [5–7]. Further, therapeutic effect of CS may be observed within 8 weeks [8]. However, long-term use of CS, especially at high doses, is associated with potentially serious adverse effects, including osteoporosis, weight gain, skin atrophy, impaired glucose tolerance, mood disorders, Cushingoid appearance and an increased risk of infection [1, 4, 9, 10]. These adverse effects can, and often do, severely impact patients’ quality of life (QoL) [4, 11].

Due to these adverse events, in clinical practice, reduction or discontinuation of CS is considered a therapeutic goal for patients with MG [12, 13]. A gradual decrease in CS dose over time is referred to as CS tapering [8]. A reduced dose of CS can also be achieved via addition of steroid-sparing therapies such as azathioprine and other NSISTs [8]. However, as with CS, NSISTs are associated with long-term side effects such as hepatotoxicity, renal toxicity, anemia, and increased risk of infection and malignancy [14–16]. Additionally, most NSISTs can take a long time to bring about the desired improvement [14]. Therefore, novel fast-acting treatment options with fewer adverse effects that can be used instead of CS or NSISTs may be highly valuable in helping patients with generalized MG (gMG).

Zilucoplan is a small macrocyclic peptide complement component 5 (C5) inhibitor with a dual mechanism of action; it prevents complement C5 cleavage to C5a and C5b and hinders the formation of C5b6 complex, should any C5b be formed, thereby preventing activation of the terminal complement pathway and formation of the membrane attack complex [2, 17]. In the 12-week Phase 3 RAISE study, zilucoplan showed statistically significant and clinically meaningful improvements in MG-specific outcomes versus placebo in a broad population of patients with anti-acetylcholine receptor antibody-positive (anti-AChR Ab +) gMG [18]. Zilucoplan had a rapid onset of action, with improvements beginning as early as one week after the start of therapy. The improvements in symptoms demonstrated in RAISE have been sustained through to 120 weeks as shown by an interim analysis of the RAISE-XT open-label extension (OLE) study in which patients were exposed to zilucoplan for up to 5.6 years, with zilucoplan also demonstrating a favorable long-term safety profile [19]. Furthermore, reduction and discontinuation of concomitant CS have been observed among patients at Week 60 [20]. We therefore conducted a post hoc interim analysis of the RAISE-XT study to evaluate the changes in CS and NSIST dose during treatment with zilucoplan up to 120 weeks. A plain language summary of this manuscript is available in Online Resource 1.

## Methods

### Patients and study design

RAISE-XT (NCT04225871) is an ongoing, multicenter, OLE study to assess the long-term safety and efficacy of zilucoplan in patients with gMG. Patients who completed the 12-week double-blind treatment period in either the Phase 2 (NCT03315130) [21] or Phase 3 (RAISE; NCT04115293) [18] studies of zilucoplan could opt to enter RAISE-XT where they self-administered daily subcutaneous injections of zilucoplan 0.3 mg/kg [20]. Patients self-administered zilucoplan at home at approximately the same time each day via a single-use portable syringe that is stable at room temperature. Full study designs of the Phase 2 study, RAISE and RAISE-XT studies, as well as full inclusion and exclusion criteria for the Phase 2 and RAISE studies, have been described previously [18, 20, 21]. In summary, patients were aged ≥ 18 years with Myasthenia Gravis Foundation of America (MGFA) Disease Class II–IV anti-AChR Ab + gMG and a Quantitative Myasthenia Gravis (QMG) score of ≥ 12. The RAISE study also required patients to have a Myasthenia Gravis Activities of Daily Living (MG-ADL) score of ≥ 6 [18]. All patients were required to be vaccinated against *Neisseria meningitidis* [20].

### Assessments

During the Phase 2 study, the RAISE study and first 12 weeks of RAISE-XT, CS and NSIST doses were kept stable as per the protocol unless medically indicated changes became necessary. Thereafter, CS and NSIST dose could be changed at the investigator’s discretion, with no criteria or schedule for dose reduction specified. The dose of CS at double-blind study baseline and changes in CS dose (discontinuation, reduction or increase), MG-ADL score and QMG score relative to double-blind baseline at Week 60 and Week 120 were assessed. Permitted CS included prednisone, dexamethasone, methylprednisone, methylprednisone sodium succinate, prednisolone, methylprednisolone and hydrocortisone.

The proportion of patients who discontinued, reduced or increased dose for ≥ 1 NSIST relative to double-blind baseline, and the impact on their MG-ADL and QMG scores at Week 60 and Week 120 were also assessed. Permitted NSISTs included azathioprine, mycophenolate mofetil/mycophenolic acid, ciclosporin/cyclosporine, cyclophosphamide, methotrexate and tacrolimus.

Safety was assessed by the incidence of treatment-emergent adverse events (TEAEs; primary endpoint of RAISE-XT). The data cutoff date for this post hoc interim analysis was November 11, 2023.

Additional assessments included time to first reduction of CS in patients on CS at double-blind baseline, time to crossing the Cushing threshold in patients who had a CS dose of ≥ 7.5 mg/day at double-blind baseline. The Cushing threshold is defined as the individual steroid dose that, if exceeded over a prolonged period, may lead to Cushing’s syndrome; it is generally considered to be a prednisone-equivalent daily dose of 7.5 mg [22–24]. Transitions from double-blind baseline to Week 120 for various daily CS dose categories up to > 30 mg/day were also assessed.

### Statistical analyses

All analyses were descriptive. Data from the modified intention-to-treat population, which included all enrolled patients in RAISE-XT who received at least one dose of zilucoplan and had at least one post-dosing MG-ADL score, were used for the CS and NSIST analyses. Data were pooled for patients in RAISE-XT who had received placebo (placebo/zilucoplan 0.3 mg/kg) or zilucoplan 0.3 mg/kg (zilucoplan 0.3 mg/kg/0.3 mg/kg) in their qualifying double-blind study. Patients who received zilucoplan 0.1 mg/kg during the qualifying Phase 2 study were not included in CS and NSIST analyses due to low patient numbers.

The daily CS dose was the total prednisone-equivalent daily dose calculated by converting the daily dose for each CS into a prednisone-equivalent dose using prespecified dose conversions and summing across each CS taken. Discontinuation and reduction of CS dose were assessed in patients on > 0 mg/day and ≥ 7.5 mg/day CS at double-blind baseline who had available data at Weeks 60 and 120. Time to first reduction of CS in patients on CS at double-blind baseline and time to crossing the Cushing threshold in patients who had ≥ 7.5 mg/day CS at double-blind baseline were calculated using Kaplan–Meier analysis. For each NSIST, daily dose was calculated for each day in the study from double-blind baseline. Discontinuation and reduction of NSIST dose were assessed in patients receiving ≥ 1 NSIST at double-blind baseline who discontinued or reduced ≥ 1 NSIST and had available data at Weeks 60 and 120. For CS and NSIST, dose increases were assessed in the overall pooled population.

Safety was assessed in the Safety Set, which included all patients who received at least one dose of zilucoplan in RAISE-XT, from RAISE-XT baseline up to data cutoff.

## Results

### Patients

Overall, 200 patients entered RAISE-XT; 183 patients from the pooled zilucoplan 0.3 mg/kg population were included in this analysis (Table 1). Broadly, there were no differences in the baseline demographics and characteristics between patients on > 0 mg/day and ≥ 7.5 mg/day CS at double-blind baseline (data not shown).

**Table 1:** Patient demographics and characteristics at double-blind baseline

	Zilucoplan 0.3 mg/kg (N = 183)
Age, years, mean (SD)	52.9 (15.0)
Sex, male, n (%)	83 (45.4)
MGFA disease class, n (%)	
Class II	54 (29.5)
Class III	117 (63.9)
Class IV	12 (6.6)
MG-ADL score, mean (SD)	10.3 (3.0)
QMG score, mean (SD)	19.0 (4.1)
Prior thymectomy, n (%)	88 (48.1)
Prior MG crisis, n (%)	59 (32.2)
Thymoma diagnosis, n (%)	43 (23.5)
Duration of disease, years, mean (SD)^a^	9.1 (9.9)
Baseline gMG-specific medication, n (%)	
CS	119 (65.0)
NSIST	93 (50.8)
Cholinesterase inhibitors	154 (84.2)

mITT population

*CS* corticosteroid, *(g)MG* (generalized) myasthenia gravis, *MG-ADL* Myasthenia Gravis Activities of Daily Living, *MGFA* Myasthenia Gravis Foundation of America, *mITT* modified intention-to-treat, *NSIST* non-steroidal immunosuppressive therapy, *QMG* Quantitative Myasthenia Gravis, *SD* standard deviation

^a^From diagnosis

### CS tapering

**Fig. 1:**
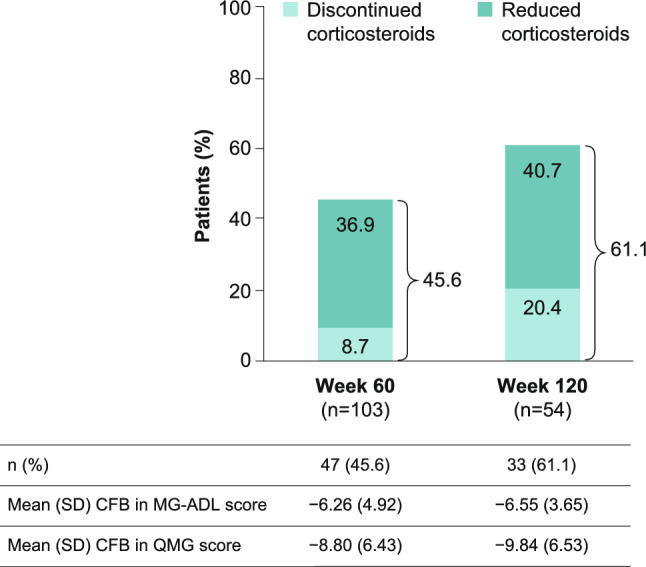
Proportion of patients who reduced or discontinued CS up to Week 120. mITT population. Data for patients with > 0 mg/day CS dose at double-blind study baseline. CFB, change from baseline; CS, corticosteroid; MG-ADL, Myasthenia Gravis Activities of Daily Living; mITT, modified intention-to-treat; QMG, Quantitative Myasthenia Gravis; *SD* standard deviation

At double-blind baseline, 119 of 183 (65.0%) patients were receiving CS, of whom 117 had dose information available. Among these patients, the mean dose of CS was 18.83 mg/day. At Week 60, 45.6% of patients who were on CS at double-blind baseline and had data available had reduced or discontinued CS. For these patients, mean CS dose at double-blind baseline was 22.3 (standard deviation [SD] 13.0) mg/day, which reduced to 9.3 (SD 8.1) mg/day at Week 60, a mean CS dose reduction of 13.0 (SD 10.4) mg/day. At Week 120, 61.1% of patients who were on CS at double-blind baseline and had data available had reduced or discontinued CS (23.0 [SD 11.6] mg/day mean CS dose at double-blind baseline to 7.5 [SD 7.8] mg/day; mean CS dose reduction of 15.5 [SD 10.2] mg/day (Fig. 1)).

**Fig. 2:**
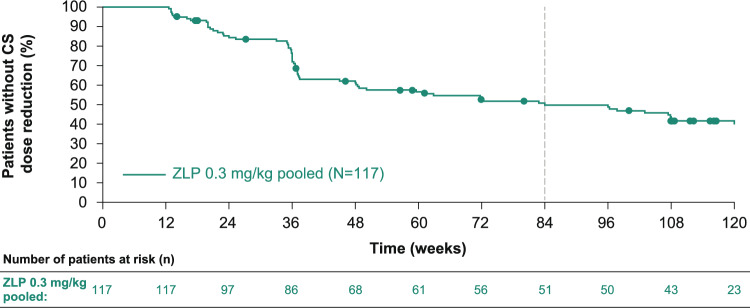
Time to first reduction of CS dose in patients with a CS dose > 0 mg/day from double-blind baseline. mITT population. The dotted line represents median time to first reduction. Patients who did not experience CS reduction were censored at the date of withdrawal/study completion or the date of their last visit. *CS* corticosteroid, *mITT* modified intention-to-treat, *ZLP* zilucoplan

The median time from double-blind baseline to first reduction of CS dose was 84.0 weeks (95% confidence interval [CI] 48.7, 120.0), with 28.1% of patients having reduced their CS dose by Week 36 (12 weeks after dose changes were permitted; Fig. 2). The median time to first reduction of CS dose was longer in patients who received placebo during the double-blind studies (96.3 weeks [95% CI 48.0, NA]; n = 52) compared with those who received zilucoplan (82.9 weeks [95% CI 45.0, 131.9], n = 65).

**Fig. 3:**
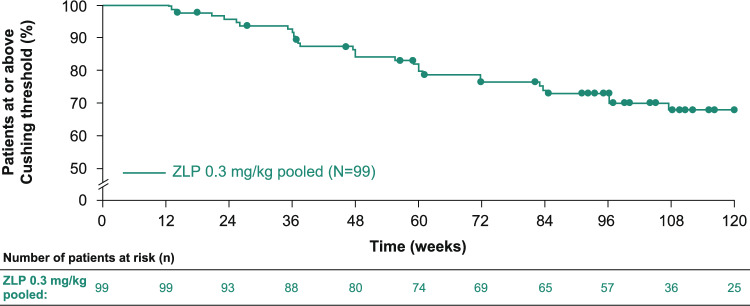
Time to crossing the Cushing threshold in patients with a CS dose ≥ 7.5 mg/day at double-blind baseline. mITT population. Patients who did not cross the Cushing threshold were censored at the date of withdrawal/study completion or the date of their last visit. *CS* corticosteroid, *mITT* modified intention-to-treat, *ZLP* zilucoplan

**Fig. 4:**
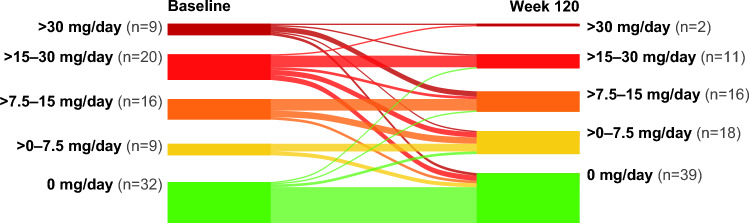
Transitions for daily CS dose categories from double-blind baseline to Week 120. mITT population. This figure demonstrates the transitions of patients between daily CS dose categories at double-blind baseline and Week 120. Only patients with observations at both timepoints are included. *CS* corticosteroid, *mITT* modified intention-to-treat

Of patients who were on ≥ 7.5 mg/day CS dose at double-blind baseline, 47.7% (n = 41/86) and 67.4% (n = 31/46) had reduced or discontinued CS by Week 60 and Week 120, respectively. Based on Kaplan–Meier analysis, at Week 60, 20.2% of patients on this dose at double-blind baseline had reduced their dose to below 7.5 mg/day, which increased to 31.9% of patients at Week 120 (Fig. 3). Of patients who were on high doses of > 15 mg/day at double-blind baseline (> 15–30 mg/day and > 30 mg/day dose categories), 58.6% (n = 17/29) reduced their dose to < 15 mg/day at Week 120 (0 mg, > 0–7.5 mg/day and > 7.5–15 mg/day dose categories; Fig. 4), with 37.9% (n = 11/29) of patients reducing their dose to ≤ 7.5 mg/day.

Among all patients with available data at Week 60 and Week 120, 4.5% (n = 7/156) and 9.3% (n = 8/86) of patients, respectively, had increased or started CS relative to double-blind baseline (mean dose increase: 13.2 [SD 6.9] mg/day at Week 60 and 11.6 [SD 9.8] mg/day at Week 120). Of these patients, 2.6% (n = 4/156) at Week 60 and 4.7% (n = 4/86) at Week 120 had started CS. In patients who increased or started CS, mean change from baseline (CFB) in MG-ADL score was −5.86 (SD 5.79) at Week 60 and −7.38 (SD 4.57) at Week 120, which was similar to those who reduced or discontinued CS (Fig. 1). Mean CFB in QMG score in patients who increased or started CS was − 7.67 (SD 2.58) and −10.14 (SD 6.20) at Week 60 and 120, respectively.

### NSIST changes

**Fig. 5:**
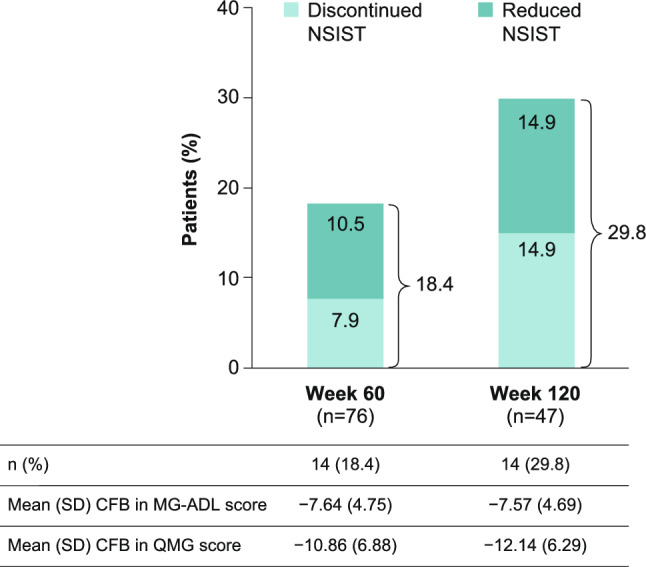
Proportion of patients who reduced or discontinued ≥ 1 NSIST up to Week 120. mITT population. Data for patients who were on ≥ 1 NSIST at double-blind study baseline. In total, 78.9% of patients at Week 60 and 68.1% patients at Week 120 had no dose changes to an existing NSIST. *CFB* change from baseline, *MG-ADL* Myasthenia Gravis Activities of Daily Living, *mITT* modified intention-to-treat, *NSIST* non-steroidal immunosuppressive therapy, *QMG* Quantitative Myasthenia Gravis *SD* standard deviation

At Week 60 and Week 120, 18.4% and 29.8% of patients, respectively, had reduced NSIST dose or discontinued ≥ 1 NSIST (Fig. 5). At double-blind baseline, 1.1% (n = 2/183) of patients were on more than one NSIST. Only 2.4% (n = 2/85) of patients increased their NSIST dose at Week 120; this included one patient who started a new NSIST (mycophenolate mofetil) at Week 72. Mean CFB in MG-ADL score in patients who reduced or discontinued NSIST (Fig. 5) was similar to those who increased or started NSIST at Week 60 and 120 (−8.00 [SD 5.66] and −6.50 [SD 4.95], respectively). Similarly, there were no meaningful differences in mean CFB in QMG score in patients who reduced or discontinued NSIST (Fig. 5) and those who increased or started NSIST (Week 60: − 10.50 [SD 2.12] and Week 120: −11.50 [SD 0.71]).

There were 32 patients with available data at Week 120 who were receiving both CS and ≥ 1 NSIST at double-blind baseline; 25.0% (n = 8/32) reduced or discontinued CS and ≥ 1 NSIST. Mean CFB in MG-ADL and QMG scores for these patients was −6.75 (SD 5.15) and −12.63 (SD 8.42), respectively.

### Safety

Safety data for the overall RAISE-XT population (N = 200) have been described elsewhere [20]. Briefly, at the data cutoff date, over a median (range) exposure of 2.2 (0.1–5.6) years, TEAEs had occurred in 97.0% (n = 194/200) of patients. The two most frequently reported TEAEs were COVID-19 (35.5% [n = 71/200] of patients) and MG worsening (29.5% [n = 59/200] of patients). In total, 40.5% (n = 81/200) of patients experienced a serious TEAE. Six serious TEAEs occurring in 2.5% (n = 5/200) of patients were considered treatment related; one (0.5%) event each of esophagitis, injection site infection (occurring on the right inner thigh, which is not a recommended injection site), colonic abscess and cellulitis across four patients, and events of headache and photophobia in one patient.

## Discussion

This post hoc interim analysis of the RAISE-XT study demonstrated that treatment with zilucoplan facilitated decrease or discontinuation of CS and NSIST in patients with anti-AChR Ab + gMG, while maintaining efficacy for up to 120 weeks. Despite their relatively rapid effect, CS often have long-term side effects, leading to a substantial treatment burden, especially at high doses [1, 4]. Similarly, NSISTs may be associated with long-term toxicities [14–16]. At Week 120, treatment with zilucoplan allowed more than 60% of patients to reduce or discontinue CS, with approximately 30% of patients able to reduce or discontinue NSISTs, which may be beneficial for managing the potential safety concerns linked with prolonged CS and NSIST use.

Despite there being no standard guidelines for the reduction of CS dose in the treatment of MG, there are some recommendations on CS use [3, 22, 25, 26]. The international consensus guidelines recommend that CS dose is gradually tapered once treatment goals have been achieved [3]. The German guidelines for the management of MG recommend that steroid-sparing strategies should be used at an early stage in the disease course [22]. Similarly, the Japanese guidelines state that low-dose steroid therapy (5 mg/day or lower) should be initiated early, with concomitant use of fast-acting treatments, such as plasma exchange and intravenous immunoglobulin, to allow for the reduction of steroid dose as part of an early fast-acting treatment strategy [26]. Guidance for the reduction of NSISTs is limited, with recommendations that their use should be tapered slowly (e.g. 500 mg/day every 12 months for mycophenolate mofetil) following achievement of treatment goals and maintenance of disease stability for a minimum of 6 months [27].

Targeted therapies such as C5 inhibitors and neonatal Fc receptor antagonists have demonstrated the potential for sustained improvements in the clinical manifestations of MG that may in turn facilitate CS tapering or discontinuation [20, 28–31]. This could prove valuable as it is known that steroid use is associated with decreased QoL [11]. Further, targeted therapies have advantages over CS and NSISTs as they have more favorable adverse event profiles, in addition to faster onset of action versus the latter [1, 15, 28]. The C5 inhibitors ravulizumab and eculizumab have both reported reductions in steroid use in patients with gMG in their OLE studies [30, 31].

In line with these observed steroid-sparing effects, our analyses of data from the RAISE-XT study show that reduction or discontinuation of CS with maintained efficacy was achieved with zilucoplan treatment. Around one-third of patients on a CS dose ≥ 7.5 mg/day at double-blind baseline reduced their dose to less than 7.5 mg/day during treatment with zilucoplan in RAISE-XT at Week 120. A 7.5 mg prednisone-equivalent daily dose is used to define the Cushing threshold, which refers to the individual steroid dose that, if exceeded, may lead to Cushing’s syndrome [22, 24]. German guidelines for the management of MG discourage long-term use of steroids above the Cushing threshold [22]. Further, it has been reported that patients receiving a CS dose ≥ 10 mg/day may have a higher adverse event burden than those on lower doses of CS [32]. Our analyses showed that zilucoplan allowed more than one-third of patients receiving a high CS dose at baseline (> 15 mg/day) to reduce their CS dose to below the Cushing threshold by Week 120, suggesting the potential for zilucoplan to reduce the treatment burden associated with CS use.

Zilucoplan also facilitated reduction or discontinuation in NSIST dose, with approximately a quarter of the patients receiving both CS and NSIST being able to reduce both of their doses. Additionally, less than 10% of patients increased or started CS or NSIST during the two-year follow-up, with only one patient requiring initiation of a new NSIST during this time, further highlighting the sustained efficacy with zilucoplan treatment.

Treatment with immunosuppressive therapies can cause non-specific immunosuppression that results in unwanted adverse effects, including an increased risk of infection [1, 15]. The use of CS is estimated to increase the risk of infection by 20–50% in patients with autoimmune diseases [33]. In patients with MG, infections have been reported to cause exacerbations, highlighting the need for targeted therapeutic approaches [33]. In this study, zilucoplan was generally well tolerated and had a favorable safety profile. Aside from the high treatment burden stemming from adverse effects, CS use in MG is also linked with an economic burden. In a 2024 United States–based study using a combined machine learning and regression approach, the number of days on CS was shown to be one of the most important predictors of high follow-up cost in patients with gMG [34].

A limitation of this post hoc analysis of the RAISE-XT study is that dose changes were at the discretion of the investigator. Investigators were neither prompted nor encouraged to reduce the CS dose hence it is possible that the estimates presented in this study may be conservative. Additional limitations include the absence of a placebo comparator due to the open-label nature of this study.

## Conclusions

To summarize, patients self-administering daily injections of zilucoplan for up to 120 weeks were able to reduce or discontinue concomitant CS and NSIST while maintaining improvement in gMG symptoms. These data highlight that treatment with zilucoplan may be beneficial for managing the safety risks associated with the long-term use of CS and NSIST.

## Supplementary Information

Below is the link to the electronic supplementary material.

Supplementary file1 (DOCX 68 KB)

## Data Availability

Underlying data from this manuscript may be requested by qualified researchers 6 months after product approval in the US and/or Europe, or global development is discontinued, and 18 months after trial completion. Investigators may request access to anonymized individual patient-level data and redacted trial documents which may include: analysis-ready datasets, study protocol, annotated case report form, statistical analysis plan, dataset specifications and clinical study report. Prior to use of the data, proposals need to be approved by an independent review panel at www.vivli.org and a signed data-sharing agreement will need to be executed. All documents are available in English only, for a pre-specified time, typically 12 months, on a password-protected portal.
