# Supplementary material for: Changes in corticosteroid and non-steroidal immunosuppressive therapy with long-term zilucoplan treatment in generalized myasthenia gravis
Source: J Neurol. 2025 Jun 12;272(7):457. doi: 10.1007/s00415-025-13113-0 (PMC12162732; doi:10.1007/s00415-025-13113-0)
Supplement: Supplementary file 1 — Supplementary file1 (DOCX 68 KB) [file 415_2025_13113_MOESM1_ESM.docx]

Supplementary information

Changes in Corticosteroid and Non-Steroidal Immunosuppressive Therapy With Long-Term Zilucoplan Treatment in Generalized Myasthenia Gravis

Channa Hewamadduma^1,2^, Miriam Freimer^3^, Angela Genge^4^, M. Isabel Leite^5^, Kimiaki Utsugisawa^6^, Tuan Vu^7^, Babak Boroojerdi^8^, Fiona Grimson^9^, Natasa Savic^10^, Mark Vanderkelen^11^, James F. Howard Jr.^12^, on behalf of the RAISE-XT study team

^1^Academic Neuromuscular Unit, Sheffield Teaching Hospitals NHS Foundation Trust, Sheffield, UK; ^2^Sheffield Institute for Translational Neurosciences (SITraN), University of Sheffield, Sheffield, UK; ^3^Department of Neurology, The Ohio State University Wexner Medical Center, Columbus, OH, USA; ^4^Clinical Research Unit, The Montreal Neurological Institute, Montreal, QC, Canada; ^5^Nuffield Department of Clinical Neurosciences, University of Oxford, Oxford, UK; ^6^Department of Neurology, Hanamaki General Hospital, Hanamaki, Japan; ^7^Department of Neurology, University of South Florida Morsani College of Medicine, Tampa, FL, USA; ^8^UCB, Monheim, Germany; ^9^UCB, Slough, UK; ^10^UCB, Bulle, Switzerland; ^11^UCB, Brussels, Belgium; ^12^Department of Neurology, The University of North Carolina at Chapel Hill, Chapel Hill, NC, USA

Corresponding author:

Dr. Channa Hewamadduma

Email: [chewamadduma1@sheffield.ac.uk](mailto:chewamadduma1@sheffield.ac.uk)

**Target Journal**: *Journal of Neurology*

# Online Resource 1 Plain language summary

Corticosteroids and non-steroidal immunosuppressive therapy (NSIST) are used as standard treatments in myasthenia gravis (MG). However, their use can lead to many side effects. Zilucoplan is a treatment for MG that improved symptoms in patients with MG in a Phase 3 clinical trial (RAISE). Patients who completed RAISE, or an earlier zilucoplan Phase 2 trial, could enter the long-term RAISE-XT trial to receive zilucoplan. During the Phase 2 trial, RAISE and the first 12 weeks of RAISE-XT, the dose of corticosteroids and NSISTs could not be changed for patients who were also receiving these treatments. After Week 12 in RAISE-XT, the investigator could change the dose. This study looked at whether treatment with zilucoplan allowed investigators to reduce the corticosteroid and NSIST dose of their patients. We assessed patients in RAISE-XT who changed their dose of corticosteroids and NSISTs compared to the start of the Phase 2 trial or RAISE.

After up to 120 weeks of treatment with zilucoplan, over 60% of patients had either reduced their corticosteroid dose or stopped corticosteroids. Additionally, about one-third of patients reduced their NSIST dose or stopped NSISTs. Even with reductions in corticosteroid and NSIST doses, the improvements in MG symptoms observed with zilucoplan treatment were maintained. Only 9% of patients increased their corticosteroid dose, and 2% increased their NSIST dose.

Our study showed that treatment with zilucoplan resulted in reduction or discontinuation of corticosteroids and NSISTs while maintaining improvement in MG symptoms. These results show that zilucoplan may be beneficial for treating people with MG experiencing negative side effects with corticosteroids and NSISTs.
